# Total Performance of Magneto-Optical Ceramics with a Bixbyite Structure

**DOI:** 10.3390/ma12030421

**Published:** 2019-01-30

**Authors:** Akio Ikesue, Yan Lin Aung, Shinji Makikawa, Akira Yahagi

**Affiliations:** 1 World-Lab. Co., Ltd., Mutsuno, Atsutaku, Nagoya 456-0023, Japan; poly-ikesue@s5.dion.ne.jp; 2 Shin-Etsu Chemical Co., Ltd., Advanced Functional Materials Research Center, Matsuida, Annaka, Gunma 379-0224, Japan; s_makikawa@shinetsu.jp (S.M.); yahagi@shinetsu.jp (A.Y.)

**Keywords:** faraday rotator material, optical isolator, transparent ceramics

## Abstract

High-quality magneto-optical ceramics (Tb*_x_*Y_1−*x*_)_2_O_3_ (*x* = 0.5–1.0) with a Bixbyite structure were extensively investigated for the first time. The total performances of these ceramics were far superior to those of commercial TGG (Tb_3_Ga_5_O_12_) crystal, which is regarded as the highest class of Faraday rotator material. In particular, the Verdet constant of Tb_2_O_3_ (when *x* = 1.0) ceramic was the largest—495 to 154 rad·T^−1^·m^−1^ in the wavelength range of 633 to 1064 nm, respectively. It was possible to further minimize the Faraday isolator device. The insertion loss of this ceramic was equivalent to that of the commercial TGG single crystal (0.04 dB), and its extinction ratio reached more than 42 dB, which is higher than the value for TGG crystal (35 dB). The thermal lens effect (1/f) was as small as 0.40 m^−1^ as measured by a 50 W fiber laser. The laser damage threshold of this ceramic was 18 J/cm^2^, which is 1.8 times larger than that of TGG, and it was not damaged during a power handling test using a pulsed laser (pulse width 50 ps, power density 78 MW/cm^2^) irradiated at 2 MHz for 7000 h.

## 1. Introduction

In 1995, highly efficient laser oscillation using a polycrystalline Nd:YAG (Y_3_Al_5_O_12_) ceramic material was reported for the first time [1]. Since then, research and development on various types of ceramic laser materials and laser oscillation has been successively reported [2]. Generally, ceramic materials are easily influenced by Mie scattering and Rayleigh scattering [3,4,5,6] as they contain many grain boundaries which degrade the oscillation efficiency and laser beam quality when they are used as a laser gain medium. However, recent studies have revealed that certain types of ceramic materials can provide novel characteristics that cannot be achieved in single crystals [7,8,9]. Polycrystalline ceramics are anticipated to be widely applied to the field of photonics in addition to laser applications. 

Faraday elements (Faraday rotator materials) that apply Faraday’s effect are classified into either Fe-containing magnetic material [10,11] or paramagnetic material [12]. The former is represented by Bi-doped iron garnet and is commonly used in isolator applications owing to its high isolation performance. The latter—paramagnetic material—has been applied for wavelength regions lower than 1.2 µm where iron garnet cannot be used. Compared with those of the Fe-included magnetic type, paramagnetic type isolators have extremely small Verdet constants. Even the commercially widely used TGG (Tb_3_Ga_5_O_12_) material with a Garnet structure has a low Verdet constant at only around 36–40 rad·T^−1^·m^−1^ [13]. Consequently, the device needs to be of length 20 mm or more, and a larger magnetic field is necessary for isolator applications. The technical issues above still need to be solved. Although NTF (Na_0.37_Tb_0.63_F_2.26_) for high-power applications [14,15] and another garnet material, TSAG (Tb_3_Sc_2_Al_5_O_12_), have been reported [16], their Verdet constants are no more than 0.7 times and 1.3 times that of TGG, respectively. 

In the case of the non-magnetic-type Faraday rotator, firstly, we considered that the content of Tb ions must be as high as possible in the materials and the crystal structure must be Bixbyite in order to improve the performance of the Faraday rotator materials, especially in terms of the Verdet constant. Additionally, we found out that the development of Tb_2_O_3_-Re_2_O_3_ (Re:Sc, Y, Lu, Gd, etc.) would be the most effective approach. However, since Tb_2_O_3_ is not stable at room temperature, when Tb_4_O_7_ is used as a starting material, (1) oxygen gas is released during the sintering process, i.e., Tb_4_O_7_ → 2Tb_2_O_3_ + 1/2O_2_, and the powder compact is finally decomposed into pieces; and (2) it appears to undergo phase transition from an orthorhombic ⇔ cubic crystal system near 1400 °C to an orthorhombic ⇔ hexagonal crystal system near 2100 °C [17,18]. These phase transitions cause volumetric changes in the material and induce mechanical stress, causing cracking, etc. Therefore, there is almost no possibility to produce this type of material using a conventional fabrication method such as melt growth or sintering technologies.

In this study, we investigated the technological difficulties in the fabrication of this material and systematically solved the above technical issues. High-quality transparent (Tb _*x*_Y_1−*x*_)_2_O_3_ (*x* = 0.5– 1.0) sintered bodies (hereafter abbreviated as TYO) with a Bixbyite structure were successfully produced, and their total performances relating to their use in practical application as a Faraday rotator device were extensively characterized for the first time. These TYO ceramics have optical properties well suited for Faraday rotators, and due to their large Verdet constants, these new materials are very promising for the development of Faraday rotators with very compact and small size compared to the commercial TGG optical isolator.

## 2. Experimental Procedures

Tb_4_O_7_ (Shin-Etsu, Tokyo, Japan, RU, 99.99%) and Y_2_O_3_ (Shin-Etsu, RU, 99.999%) powders were used as starting materials. Tb_2_O_3_ (yellowish white color) powders were prepared by heat treating the dark brown Tb_4_O_7_ raw powders (Shin-Etsu, RU, 99.99%) under a hydrogen atmosphere. Ethanol (analytical reagent) was used as the solvent. A small amount of ZrO_2_ (TOSOH, Tokyo, Japan, TZ-0, 99.9%: 0.5–1.5 mass %) was used as a sintering aid.

Tb_2_O_3_ powder was mixed with Y_2_O_3_ powder in ethanol solvent for 10 h by a conventional ball-milling process. The obtained slurry was dried and granulated by using a spray-dryer (SAKAMOTO Engineering, TRS-4W, Kawasaki, Japan). The premixed Tb_2_O_3_-Y_2_O_3_ powders were made into tablets using a metal mold (internal diameter: 8 mm) by uniaxial pressing (RIKEN, CDM-5PA, Tokyo, Japan). Then, the tablets were isostatically pressed in a CIP machine (cold isostatic press, KOBE Steel, P200, Tokyo, Japan) with a pressure of 196 MPa. Depending on the Tb doping level, these powder compacts were sintered in a vacuum furnace (W-heater, Special ordered furnace, Futek Furnace Inc., Yokohama, Japan) under a vacuum level of 1 × 10^−3^ Pa at 1500–1680 °C for 3 h. Then, the pre-sintered tablets were treated in an HIP (hot isostatic press, KOBE Steel, SYS50X-SB, Tokyo, Japan) machine with a temperature range from 1500 to 1700 °C for 2 h under Ar gas pressure at 176 MPa. The sintering temperature and pressure were adapted in accordance with the Tb content. Transparent ceramics were achieved after the HIP treatment. Their basic optical properties and magneto-optical properties were investigated. Details of the characterization are described in the Supplementary Materials. The technical issues relating to single-crystal TYO grown by the conventional melt growth method are also discussed in the Supplementary Materials.

To evaluate the Faraday rotation performance, the same experimental setup reported in a previous paper was used [19]. Samples of (Tb_0.6_Y_0.4_)_2_O_3_ ceramics (5 mm in diameter by 8 mm length) and TGG single crystal (5 mm in diameter by 20 mm length, Electro-Optics Technology Inc., Traverse City, MI, USA) with <111> orientation were used. Each sample was clamped in a copper holder and put in a commercial Faraday rotator magnetic housing. The average magnetic field exerted on the TGG crystal and (Tb_0.6_Y_0.4_)_2_O_3_ ceramics was 1 T. A polarization plane of laser light was rotated by the Faraday effect due to the magnetic field. The transmitted laser output was measured using a power meter.

## 3. Results

### 3.1. Synthesis and Characterization of the Novel Ceramic Faraday Rotator Material

A ceramic fabrication process was applied to produce sintered bodies with a (Tb_0.6_Y_0.4_)_2_O_3_ composition. Tb_4_O_7_ (Shin-Etsu, RU, 99.99%) and Y_2_O_3_ (Shin-Etsu, RU, 99.999%) powders were used as starting materials; they were mixed in ethanol solvent for 10 h, then the dried premixed powders were pressed in a CIP (Cold Isostatic Press) machine at 196 MPa. The color of the obtained powder compacts was dark brown. Then, the powder compacts were sintered (1) under hydrogen atmosphere and (2) under vacuum (1 × 10^−3^ Pa) at 1600 °C for 2 h, separately. In both processes, almost all samples were crushed into pieces after sintering in hydrogen, and many cracks occurred after sintering in vacuum. 

From the TG-DTA (Thermogravimetry Differential Thermal Analysis, Thermo plus EVO TG8120, RIGAKU, Akishima, Japan) analysis result, we concluded that the following oxygen dissociation reaction occurred during the heating of Tb_4_O_7_ due to the release of oxygen inside the material when the samples cracked or were crushed into pieces.

7Tb_4_O_7_ ⇔ 4Tb_7_O_12_ + 1/2O_2_ at 540 °C

2Tb_7_O_12_ ⇔ 7Tb_2_O_3_ + 3/2O_2_ at 940 °C

**Figure 1:**
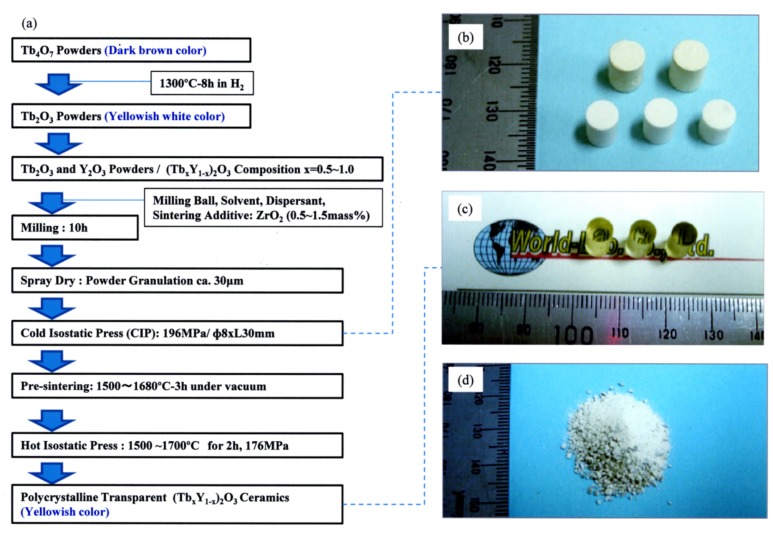
(**a**) Fabrication process for (Tb*_x_*Y_1−*x*_)_2_O_3_ ceramics derived from Tb_2_O_3_ and Y_2_O_3_ raw powders; (**b**) powder compacts after the cold isostatic press (CIP) process; (**c**) transparent sintered bodies with ZrO_2_ additives after hot isostatic press (HIP) treatment; (**d**) crushed sintered bodies without ZrO_2_ additives after sintering.

To avoid this cracking problem, first, Tb_2_O_3_ (yellowish white color) powders were prepared by heat treating the dark brown Tb_4_O_7_ raw powders (Shin-Etsu, RU, 99.99%) under a hydrogen atmosphere. The fabrication process for TYO ceramics derived from Tb_2_O_3_ is shown in Figure 1a. Then, the Tb_2_O_3_ powder was mixed with Y_2_O_3_ (Shin-Etsu, RU, 99.999%) powder in ethanol solvent for 10 h by a ball-milling process. The obtained slurry was dried and granulated using a spray-dryer. The premixed Tb_2_O_3_-Y_2_O_3_ powders were made into tablets using a metal mold (Φ 8 mm) by uniaxial pressing and a CIP (cold isostatic press) machine with a pressure of 196 MPa. The obtained powder compacts are shown in Figure 1b. Depending on the content of Tb, these power compacts were sintered under vacuum (1 × 10^−3^ Pa) conditions at 1500–1680 °C for 3 h. Then, the pre-sintered tablets were treated in an HIP (hot isostatic press) machine with a temperature range from 1500 to 1700 °C for 2 h under Ar gas pressure at 176 MPa. The sintering temperature and pressure were adapted in accordance with the Tb content. After the HIP treatment, yellowish transparent ceramics were achieved (see Figure 1c). The color of the transparent ceramic samples varied with the content of Tb ions. The higher the Tb content was, the deeper the color of the sample. However, when pure Tb_2_O_3_ was produced, it was colorless and transparent. If there were no sintering aids used, the grain growth was accelerated during sintering, and the sintered sample showed poor translucency or cracks occurred inside the samples, or it ended up cracking into pieces in the worst case (see Figure 1d).

**Figure 2:**
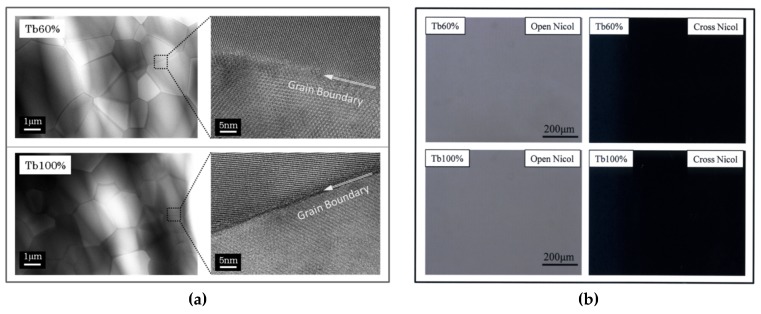
(**a**) The microstructure of (Tb_0.6_Y_0.4_)_2_O_3_ and Tb_2_O_3_ ceramics after HIP treatment as observed by TEM. (**b**) Transmission polarized optical microscopic images of the produced (Tb_0.6_Y_0.4_)_2_O_3_ and Tb_2_O_3_ ceramics.

The microstructures of the (Tb_0.6_Y_0.4_)_2_O_3_ and Tb_2_O_3_ ceramics after HIP treatment were observed by TEM (transmission electron microscopy, ARM-200F, JOEL, Tokyo, Japan), and their images are shown in Figure 2a. It was confirmed that both ceramics were composed of grains of size of the order of several µm with different crystal orientations, and neither secondary phases nor grain boundary phases were observed. The lattice structure was observed to be a Bixbyite structure towards the grain boundary regions, and a clean grain boundary was confirmed. TEM-EDS (electron dispersive spectroscopy) analysis results of inner grain and grain boundary revealed that there was no segregation of ZrO_2_, which was added as a sintering additive. Figure 2b shows the transmission polarized optical microscopic images of the (Tb_0.6_Y_0.4_)_2_O_3_ and Tb_2_O_3_ ceramics. There was no birefringence, and they were optically homogeneous. No residual pores, the main factor in optical scattering, were detected inside the materials. XRD (X-ray diffraction, X'PERT PRO MPD, Malvern Panalytical, Almelo, The Netherlands) results revealed that the crystal system was only cubic phase and there were no other phases. When these ceramics were heat treated above 1400 °C, an orthorhombic ⇔ cubic phase transition occurred and the general optical quality was degraded. As seen in the above SEM and TEM images, the microstructures of the developed TYO ceramics were of a high-quality finished form.

It was anticipated that the added ZrO_2_ would play an important role in inhibiting grain growth during the sintering of TYO ceramics; hence, the sintered bodies were composed of fine grains. Accordingly, (1) damage due to phase transition was effectively reduced by forming numerous grain boundaries, or (2) ZrO_2_ itself possibly inhibited the phase transition. In the case of TYO ceramics without the addition of ZrO_2_, the optical quality of the sample was very poor due to the significant grain growth during the sintering process or the sample having broken into pieces.

### 3.2. Optical Properties of the Advanced Materials

**Figure 3:**
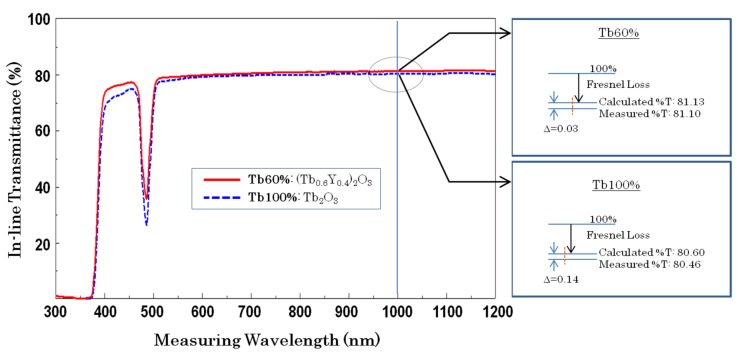
In-line transmittance curves of the (Tb_0.6_Y_0.4_)_2_O_3_ and Tb_2_O_3_ ceramics (Sample thickness = 5 mm, optical-polished surfaces).

**Figure 4:**
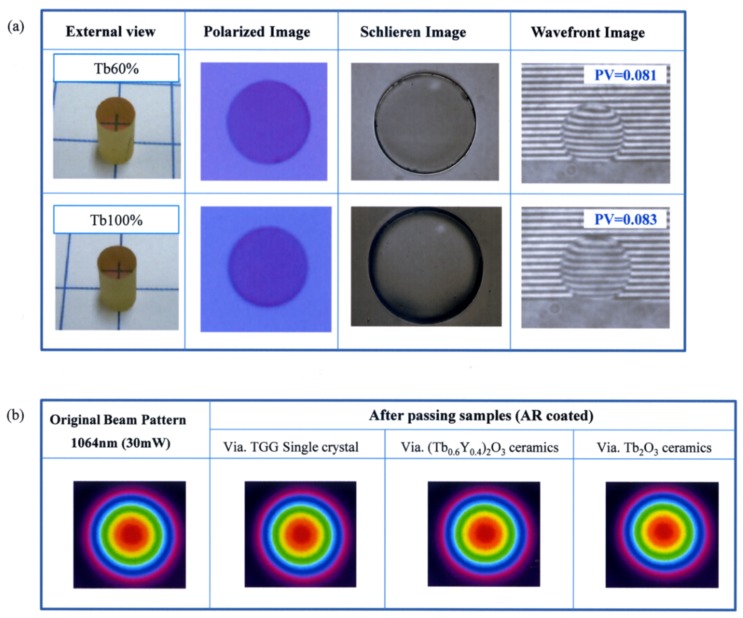
(**a**) External view, polarized image, Schlieren image, and wavefront image by interferometry of the (Tb_0.6_Y_0.4_)_2_O_3_ and Tb_2_O_3_ ceramics. (The thickness of each sample was 11 mm.) (**b**) Original beam pattern and beam patterns after passing through the TGG single crystal and the produced (Tb_0.6_Y_0.4_)_2_O_3_ and Tb_2_O_3_ ceramics.

The in-line transmittance curves of the (Tb_0.6_Y_0.4_)_2_O_3_ (Tb 60%) and Tb_2_O_3_ (Tb 100%) ceramics from the visible to near-infrared wavelength regions are shown in Figure 3. The thickness of each sample was 5 mm, and the surfaces were optical polished but without AR (anti-reflective) coating. Only absorption due to Tb^3+^ ions can be confirmed around 480 nm, and the absorption of Tb 100% was stronger than that of Tb 60%. Wavelength dependency of the transmission lines was not detected for both ceramics, suggesting no Rayleigh scattering inside the materials. The refractive indices of Tb60% and Tb 100% materials at a 1µm wavelength are 1.920 and 1.940, respectively. The surface reflection loss (Fresnel loss) at a surface against air can be calculated by the following equation:

β(λ) = [(n(λ) − 1)^2^]/[(n(λ) + 1)^2^], (1)

where β(λ) is a reflection loss and n is a refractive index. Accounting for both sides of a sample, this Fresnel loss value is doubled to obtain theoretical transmittance. Their measured transmittance values, especially at a 1 µm wavelength, were very close to the theoretical values calculated by subtracting the Fresnel loss due to surface reflection. This result indicates that their optical losses are very low. An external view, polarized image, Schlieren image, and wavefront image by interferometry of the (Tb_0.6_Y_0.4_)_2_O_3_ and Tb_2_O_3_ ceramics are shown in Figure 4a. The thickness of each sample was 11 mm. No optical inhomogeneity was observed in any inspection methods. The wavefront distortion was less than λ/10, suggesting that the ceramics are well suited for use as optical materials.

A laser with an output power of 30 mW (1064 nm wavelength, beam spot size: 2 mm) and with a TEM_00_ mode was used as a light source to evaluate the beam quality after passing through the sample. For comparison, a commercially available TGG single crystal was also measured as a reference. The original beam pattern and those after passing through the TGG single crystal and the produced (Tb_0.6_Y_0.4_)_2_O_3_ and Tb_2_O_3_ ceramics are compared and summarized in Figure 4b. The original beam pattern was in the Gaussian mode, and the beam patterns that passed through the TGG single crystal, (Tb_0.6_Y_0.4_)_2_O_3_, and Tb_2_O_3_ ceramics were almost unchanged. This result suggested that the variation of the refractive index inside the material is extremely small, which is in accordance with the measured results shown in Figure 4a. 

When a laser with an output power of 50 W laser (1070 nm wavelength, CW (continuous wave) single-mode ytterbium fiber laser manufactured by IPG photonics corp., Burbach, Germany) was used as a light source, the beam shape after passing through the (Tb_0.6_Y_0.4_)_2_O_3_ sample was slightly deformed due to the thermal lens effect (1/f = 0.40 m^−1^: change in beam waist of passed laser beam). When the same measurement method was used, the value for TGG crystal was 1/f = 0.35 m^−1^, which is slightly better than that for the (Tb_0.6_Y_0.4_)_2_O_3_ ceramics. However, for Y_2_O_3_ ceramics, which have similar optical loss, the value was 1/f = 0.34 m^−1^. As seen in Figure 1c, it is considered that a trace amount of Tb^4+^ ions remained in the (Tb_0.6_Y_0.4_)_2_O_3_ ceramics, and optical absorption by these Tb^4+^ ions caused the thermal lens effect during laser irradiation. In addition, it was also confirmed that the material was not damaged by a power handling test which is generally used for commercial isolators developed for fiber lasers. In this test, a pulsed laser (pulse width 50 ps, peak power 0.3 MW, beam spot Φ 0.7 mm, power density 78 MW/cm^2^) was irradiated at 2 MHz for 7000 h. In addition, when a laser damage test using an Nd:YAG laser with a wavelength of 1064 nm, a pulse width of 4 ms, and a laser focusing diameter of Φ 50 µm was performed, TYO ((Tb_0.6_Y_0.4_)_2_O_3_) and TGG single crystal were damaged at an average power of 18 and 10 J/cm^2^, respectively. From this result, it was also confirmed that the damage threshold of TYO ceramics is excellent, and the only technical issue is to reduce the thermal lens effect faintly generated during laser irradiation.

**Figure 5:**
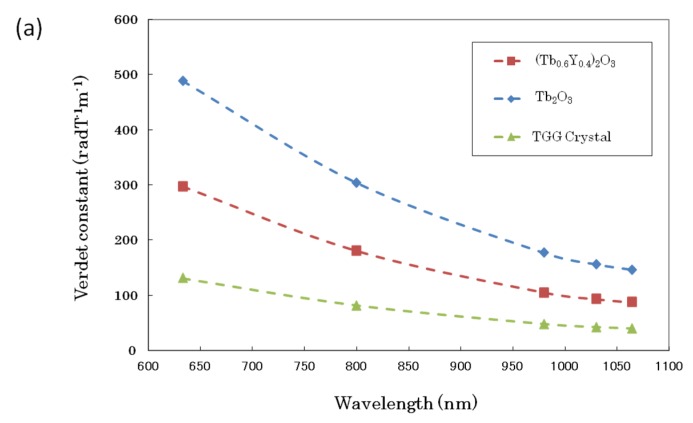

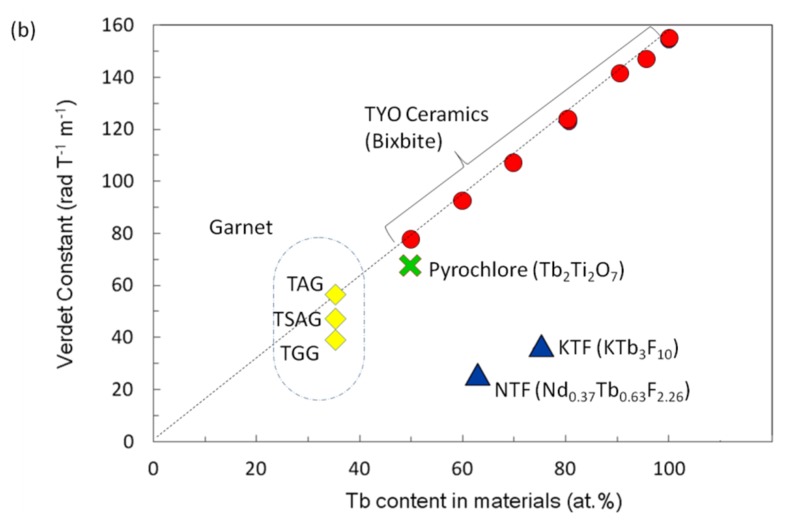
(**a**) Wavelength dependency of the Verdet constant for the TGG single crystal and the (Tb_0.6_Y_0.4_)_2_O_3_ and Tb_2_O_3_ ceramics. (**b**) Relationship between the concentration of Tb ions in (Tb_*x*_Y_1−*x*_)_2_O_3_ ceramics and Verdet constant for 1 µm wavelength.

The wavelength dependencies of the Verdet constant for the TGG single crystal and the (Tb_0.6_Y_0.4_)_2_O_3_ and Tb_2_O_3_ ceramics measured at wavelengths 633, 800, 980, 1030, and 1064 nm are shown in Figure 5a. At any wavelength, the Verdet constants of (Tb_0.6_Y_0.4_)_2_O_3_ and Tb_2_O_3_ ceramics were about two times higher that of the TGG single crystal. In particular, in the case of the Tb_2_O_3_ ceramics, the Faraday rotation angle was found to be about 3.8 times (as a maximum) higher than that of the TGG single crystal. The relationship between the concentration of Tb ions (i.e., the occupancy of Tb^3+^ ions in the total number of cations) in TYO ceramics with a Bixbyite structure and the Verdet constant for 1 µm wavelength is shown in Figure 5b. The Verdet constants of Faraday rotator materials with other crystal structures are also plotted in the same figure as a reference. The Verdet constant simply increased with increasing Tb ion concentration, and 154 rad·T^−1^·m^−1^ was achieved as the highest value. As the Verdet constant increases, the required length of the Faraday rotator can be reduced.

In the case of non-magnetic materials, the required length of the Faraday elements such as TGG single crystal and TYO ceramics to provide a 45° Faraday rotation angle depends on the strength of applied magnetic field. In the case of TGG single crystal, a length of 20 mm is required to obtain a 45° Faraday rotation angle when 1 T of magnetic field is applied. By using the ceramics with the highest Verdet constant, the length can be shortened to 5.1 mm to obtain a 45° Faraday rotation angle under 1 T of magnetic field, suggesting that the Faraday rotator device can be manufactured with a very compact size, whereas a commercial TGG crystal requires a length of about 20 mm. As seen in Figure 5b, TYO ceramics with a Bixbyite structure showed very high Verdet constants against Tb concentration (i.e., Tb ion number/total cation number) compared to other Faraday rotator materials such as TGG [13], TSAG [16], TAG [19], NTF (Na_0.37_Tb_0.63_F_2.26_) [14,15], KTF (KTb_3_F_10_) [20], and TTO (Tb_2_Ti_2_O_7_) [21]. 

**Figure 6:**
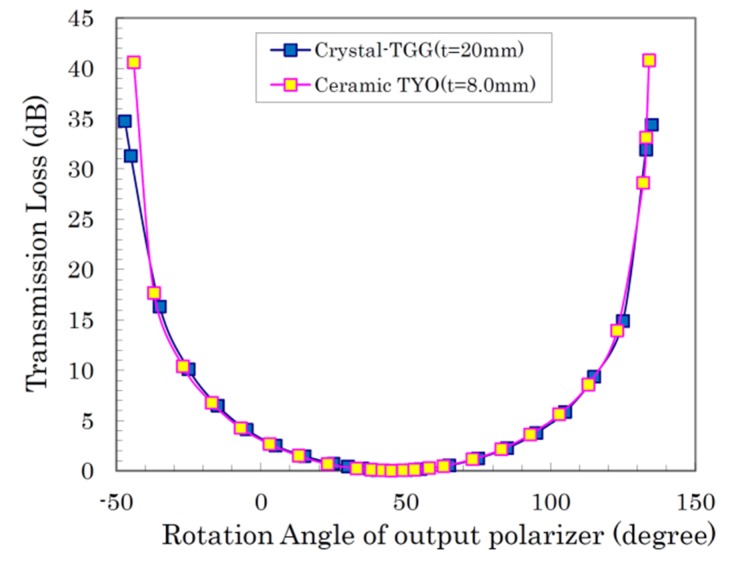
Faraday rotation characteristics of the TYO ceramics in comparison with those of the commercial TGG single crystal.

The measured results for transmitted laser power against the rotation angle of the polarizer under a 1 T magnetic field are shown in Figure 6. The Faraday rotation characteristics of the (Tb_0.6_Y_0.4_)_2_O_3_ ceramics with very high Verdet constant were analogous to those of the commercial TGG single crystal, and their transmitted laser power values at a 45° rotation angle of the polarizer were comparable to each other (insertion loss: 0.04 dB). Compared with the maximum extinction ratio (E.R.) of the TGG single crystal (E.R.: 35 dB) where the rotation angle of polarizer was at −45°, the extinction ratio for the (Tb_0.6_Y_0.4_)_2_O_3_ ceramics was as high as 42 dB. These materials with a very high Verdet constant can provide an equivalent Faraday rotation angle and a high extinction ratio even from a half-length (8.0 mm) of the conventional TGG single crystal (length: 20 mm). In other words, an advantage to replacing existing Faraday rotators with a high-Verdet-constant Faraday rotator is that if the length is kept the same size as the conventional single crystal, the required magnetic field can be reduced by more than half. In the case of Tb_2_O_3_ ceramics, principally it can be reduced to about one-fourth to obtain the same Faraday rotation performance as (Tb_0.6_Y_0.4_)_2_O_3_ ceramics. The Tb_2_O_3_ ceramic (*t* = 5.1 mm), which had the highest Verdet constant, also showed similar behavior to TGG crystal, its insertion loss (I.L.) was 0.19 dB at a 45° rotation angle of the polarizer, and its extinction ratio was 47 dB at a −45° or 135° rotation angle of the polarizer.

## 4. Discussion

The magneto-optical performance represented by the Verdet constant of Faraday rotator materials for the visible to 1.2 µm wavelength regions is not high enough since they do not include the magnetic element Fe in the composition of the materials. Although TAG [22,23,24] or TGG [13] single crystal are commonly used there, their Verdet constants are limited, and there is practically no other material which possesses a higher Verdet constant. It is very difficult to grow TAG single crystals by the CZ (Czochralski) method with acceptable aperture size because of their incongruent melting nature and unstable TAG phase in the Tb_2_O_3_-Al_2_O_3_ system [23]. The FZ (floating zone) method has often been used to grow TAG crystal, but its application is limited to research purposes only. Recently, it has been reported for TGG and TAG polycrystalline ceramics [25,26,27,28,29], but these do not exceed the conventional single-crystal Faraday rotator in terms of fundamental performance. The authors have demonstrated high-quality (Tb_1−*x*_Y*_x_*)_3_Al_5_O_12_ (TAG) ceramics showing superior optical properties than the TAG single crystals prepared by the FZ method. It is the only example that has solved the problems relating to TAG single crystals and the performance of the conventional TGG single crystal.

In order to achieve a 45 degree Faraday rotation angle using a conventional single crystal under 1 T magnetic field, the rotator material needs to be as long as 20 mm; hence, a larger magnet is needed to supply a large enough magnetic field. Basically, the value of the Faraday rotation angle of the Faraday rotator is defined by the value of the Verdet constant, and the Verdet constant is determined dominantly by the occupancy of Tb ions which contributes to the magneto-optical effect, i.e., the Ga ions in TGG do not contribute to the Faraday rotation. TAG and TGG both have a garnet structure; hence, the occupancy of Tb^3+^ ions (Tb/(Tb+Al)) in the total number of cations is limited to 37.5%. Accordingly, garnet materials are not sufficient to achieve large Faraday rotation from the viewpoint of Tb^3+^ ion occupancy. 

Based on the above viewpoint, Tb_2_O_3_ material may result in the highest occupancy of Tb^3+^ ions. However, since the melting point of Tb_2_O_3_ in its phase diagram is about 2300 °C and it has a phase transition from an orthorhombic ⇔ cubic crystal system near 1400 °C to an orthorhombic ⇔ hexagonal crystal system near 2100 °C [17,18], it is theoretically impossible to grow Tb_2_O_3_ single crystals by the conventional melt growth process. We confirmed that the polycrystalline TYO (including Tb_2_O_3_) ceramics have appropriate properties as an efficient optical isolator and also that the fabrication process is economically efficient. Hence, we filed a patent on the TYO ceramics in 2011 [30]. In 2015, light-yellow-colored Tb_2_O_3_ single crystals grown by using a Li_6_Tb(B_2_O_3_)_3_ system were reported with a low melting point flux around 1235–1160 °C, which is lower than the phase transition points [31]. However, the resulting crystal size was about 5 mm × 5 mm × 1 mm, which is too small for practical applications. Furthermore, optical quality was discussed there only by means of transmission curves, which are not enough to evaluate the opacity of thin film. The scattering loss of this material has been roughly estimated to be as large as around 10%/pass which requires a rotator length of 5.1 mm from the Verdet constant. In addition to this, it is not possible to control the crystal orientation, i.e., axis of easy magnetization, during crystal growth. Snetkov et al. synthesized Tb^3+^:Y_2_O_3_ ceramics [32] but the transparency of their material is extremely poor and it cannot be used for optical applications. Although the basic characteristics of the TYO ceramics were reported in our previous paper [33], it is important to demonstrate the total performance parameters which are required to determine it to be a practical Faraday rotator material.

On the other hand, optical-grade YAG (Y_3_Al_5_O_12_) and Sesquioxide ceramics denoted by Re_2_O_3_ (Re: lanthanide rare-earths) have been reported recently. Basically, a small amount of laser active elements is doped into the Re_2_O_3_ host materials (Re: Sc, Y, Lu) which do not have phase transition points up to 2000 °C. Then they are sintered at high temperature (over 1700 °C) to produce transparent ceramic materials. It has been confirmed that those materials are suited to laser gain media or scintillators, etc. [34,35]. It has been found that the Tb-containing materials with composition TYO can solve the technical difficulties of the conventional isolators. Here, it is necessary to increase the content of Tb ions in the solid solution to as high as possible. However, a technical issue there is that the fabrication temperature needs to be decreased with increasing Tb ion content in the solid solution. In particular, the ultimate material, Tb_2_O_3_, has very low phase transition temperature; hence, it is necessary to fabricate even Tb_2_O_3_ polycrystalline ceramics at temperatures lower than 1400 °C. In this report, we examined how we succeeded in densifying the materials to get high transparency, and finally examined the possibility of applications to optical isolators.

The question arises as to why single crystals have only been used as Faraday rotators so far. The reason for this is that optical scattering loss is small in single crystals and a uniform Faraday rotation angle can be achieved when a magnetic field is applied to a unidirectionally oriented crystal (<111> in general). It is well known that even a cubic crystal system has magneto-crystalline anisotropy. For example, in the case of TSAG (Terbium-Scandium-Aluminum Garnet) crystal, the Verdet constant of the <111> orientation, which has the smallest surface energy, is about 5–7% larger than that of the <110> or <100> orientation. [36] It is anticipated that the Verdet constants of other crystal orientations in a cubic system, which have higher surface energy, will be smaller than that of the <111> orientation. Consequently, it is likely that the Verdet constant of polycrystalline ceramics composed of numerous microcrystallites with randomized crystal orientations might be different from one grain to another, and the Faraday rotation behavior of polycrystalline ceramics might be different from that of single-crystal Faraday rotator materials. Here, the Faraday rotation angle is expressed as the equation below [37,38]:

θ_F_ = VHL, (2)

where θ_F_ is the Faraday rotation angle, V is the Verdet constant, H is the applied magnetic field, and L is the length of the Faraday rotator material.

In the case of ceramic materials, each grain has a randomized crystal orientation, and when a magnetic field is applied, the Faraday rotation angle will be slightly different in each grain of the ceramics; however, this difference did not cause any disadvantages upon utilization. When they were compared to the TGG single crystalline materials, it was confirmed that the TYO ceramics have equivalent values of the Faraday rotation angle and higher extinction ratios. In addition, this study has revealed the discovery of a novel Faraday rotator material which possesses a Verdet constant approximately 4 times higher than that of the commercial TGG single crystal. 

## 5. Conclusions

Since the Faraday effect was discovered in 1845, a wide variety of Faraday rotator materials, such as glass or single crystal, have been developed and have progressed into the practical phase with developments in the field of telecommunication and machining. Only single-crystal materials have been put into practical use until now, but this does not necessarily mean that Faraday rotator materials are only limited to single crystals. As this work demonstrated, it is also possible to create new materials with excellent performance by other inorganic material processes such as ceramic fabrication processes. We successfully produced optical-grade TYO ceramics in this work, and their total performance as a Faraday rotator is summarized below.

(1) Optical-grade polycrystalline TYO ceramics with extremely low scattering were successfully produced for the first time.
(2) The Verdet constants of the TYO ceramics increased with increasing Tb concentration in the Bixbyite structure, and Tb_2_O_3_ showed the highest value: 3.8 times higher than that of the commercially available TGG single crystal.
(3) The Faraday rotation characteristics of the polycrystalline TYO ceramics were basically comparable to those of single-crystal isolator materials. In addition, one of the advantages was the possession of a large extinction ratio and a large Verdet constant, which can improve the performance of the isolator and downsize the device.
(4) The laser damage threshold of the TYO ceramics was as high as 18 J/cm^2^ and they were resistant to pulsed laser damage (power density 78 MW/cm^2^ and no damage during a 7000-hour durability test at 2 MHz).
(5) The thermal lens value, 1/f = 0.40 m^−1^, of the TYO ceramics was slightly larger than that of TGG, probably due to a remaining trace amount of Tb^4+^ ions in the material. One of the remaining issues is to be able to use it for high-power and continuous-wave laser applications.

## Supplementary Materials

The following are available online at http://www.mdpi.com/1996-1944/12/3/421/s1, Figure S1:(a) External view, and (b) polarized optical microscopic image of (Tb_0.5_Y_0.5_)_2_O_3_ single crystal; Figure S2: Relationships between the Tb ion concentration and the refractive index, and the thermal conductivity; Figure S3: (a) Prototype of optical isolator using TYO (Tb-60%) ceramic in comparison with commercial TGG optical isolator. (b) Schematic diagram of optical isolator. (c) Magnetic flux distribution inside the magnet house of optical isolator and the position of Faraday rotator sample influenced by the magnetic field. (d) Comparison of features of each Faraday rotator material; Figure S4: Appearance of large scaled TYO ceramic samples with various aperture sizes.
