# Supplementary material for: Total Performance of Magneto-Optical Ceramics with a Bixbyite Structure
Source: Materials (Basel). 2019 Jan 30;12(3):421. doi: 10.3390/ma12030421 (PMC6384737; doi:10.3390/ma12030421)
Supplement: Supplementary file 1 [file materials-12-00421-s001.pdf]

Supplemental Materials

Total Performance of Magneto-Optical Ceramics with a Bixbyite Structure

Akio Ikesue [1], Yan Lin Aung [1,*], Shinji Makikawa [2], and Akira Yahagi [2]

[1] World-Lab. Co., Ltd., Mutsuno, Atsutaku, Nagoya 456-0023, Japan; poly-ikesue@s5.dion.ne.jp
[2] Shin-Etsu Chemical Co., Ltd., Advanced Functional Materials Research Center, Matsuida, Annaka, Gunma 379-0224, Japan; s_makikawa@shinetsu.jp (S.M.); yahagi@shinetsu.jp (A.Y.)
* Correspondence: poly-yan@r2.dion.ne.jp

Fabrication of Single Crystal TYO by FZ Method

A single crystal TYO was grown by conventional melt growth method and technical issues were discussed. An external view of $(Tb_{0.5}Y_{0.5})_2O_3$ single crystal grown by FZ method is shown in Figure S1(a). Firstly, powder compact having a composition of Tb_4O_7 (50 mol%)-Y_2O_3 (50 mol%) was sintered under Ar-3%H_2 atmosphere for 2 h at 1500 °C, and then it was grown by the FZ (floating zone) method (crystal growth rate 5 mm/h, rotation speed 30 rpm, and atmosphere Ar-8%H_2). Internal microstructure was observed under transmission polarized optical microscope (see Figure S1(b)). It was not homogeneous. Voids, cracks, double refractions and inclusions were observed in all positions of the crystal. The crystal structure of this material at room temperature is a cubic system. However, during cooling process after melting at 2400 °C phase transition occurred from hexagonal ⇒ orthorhombic ⇒ cubic crystal system. Therefore, some parts were not confirmed as dark-field under cross nicols due to the formation of optically anisotropic phases. Insertion loss (I.L.) and extinction ratio (E.R.) were measured (sample thickness: 5mm). The average values of insertion loss (I.L.) and extinction ratio (E.R.) were 2.57 dB and 10.6 dB, respectively, which imply very high optical loss and very small extinction ratio. Therefore, it is noteworthy that even a single crystal TYO produced by melt-growth method cannot provide a good optical quality with practical size for this kind of isolator material.

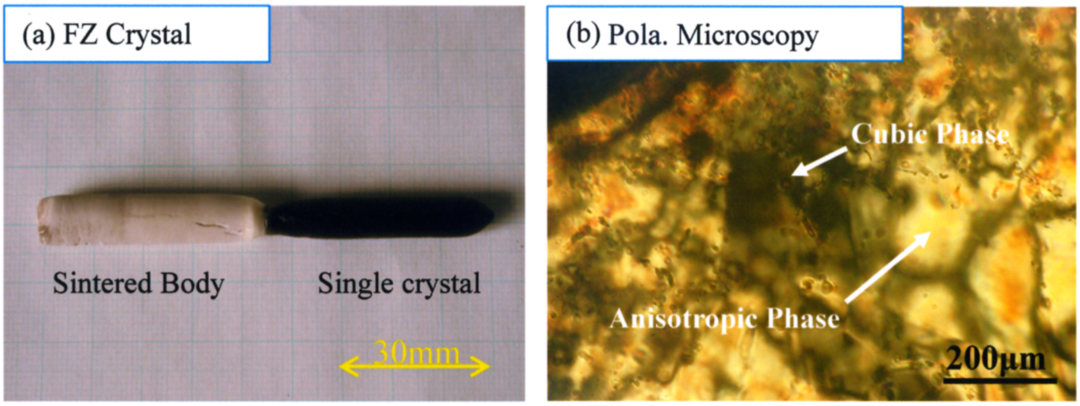

Figure S1. (**a**) External view, and (**b**) polarized optical microscopic image of $(Tb_{0.5}Y_{0.5})_2O_3$ single crystal.

Characterization on TYO Ceramics

Thermal weight analysis and differential thermal analysis (TG-DTA) of the starting powder (Tb_4O_7) were performed using Rigaku (Thermo Plus EVO TG8120) with a heating rate of 15 °C/min in flowing air. SEM images were obtained with a JEOL scanning electron microscope (JSM-7000F) operated at 10kV. TEM images were acquired with a JEOL spherical aberration corrected Scanning Transmission Electron Microscope (Cs-corrected STEM, ARM-200F) operated at 200kV. Samples for TEM analysis were prepared as follow. A sample with 3 mm diameter was cut out by ultrasonic

processing after making a thin sheet of sample by diamond polishing. Then the center part of the sample was polished down to about 20 μm by a dimpler, and finally finished up by Ar-ion milling (GATAN PIPS). In order to prevent charge-up issue, carbon deposition was done on the surface of the finished samples. Transmission polarized optical microscopic images were obtained using an Olympus BX50 attached with polarizer plate. Transmission and absorption spectra were measured by using a spectrophotometer (JASCO, V-670). Optical-polished samples with a thickness of 5mm were used. Polarized image was obtained by using a macro polarizer (TOSHIBA, SVP-200). Variations in refractive index for the whole position of each ceramic sample were observed by using a Schlieren photography system (Mizojiri Kogaku, SLM-10S). Optical wavefront distortion was measured at 632nm using an interferometer (GPI-XP, Zygo Ltd., USA). Optical polished samples with surface flatness better than $\lambda/10$ was used for this measurement. Infrared DPSS laser (Sanctity Laser, 1064 nm) with Gaussian mode was irradiated into the sample. Then the transmitted laser beam pattern was recorded on a beam profiling camera (Spiricon, SP620U).

The Verdet constant was determined by the following method. 20.0mm long TGG crystal (Electro-Optics Technology Inc.) and 8.0 mm long $(Tb_{0.6}Y_{0.4})_2O_3$ (abbr. as "TYO") ceramics were used as Faraday rotator in this measurement. The outer diameter of each sample was 5.0mm. The Faraday rotator sample was placed inside a hollow cylinder magnet (Nd-Fe-B, Shin-etsu Chem. Co.) such that the sample is located at the center of light axis. The wavelength of light source (cw laser diode, 10mW, FiberLabs Inc. FPLD-1060-24) with 1064 nm was irradiated into the Faraday rotator along the light axis. The Faraday rotation angle of the output laser beam was measured to be 45.0 degree by using polarizer plates. The distribution of magnetic field intensity was calculated by finite element method (JMAG-Designer). Magnetic field intensity applied to each sample was 0.98 T for 20 mm long TGG crystal and 1.12T for 8.0 mm long TYO ceramics, respectively. From the above measurement results, Verdet constants were determined by the following formula: $\theta_F = VHL$, where is Faraday rotation angle, H is magnetic field intensity, and L is length of the Faraday rotator.

Thermal conductivities of each ceramic sample were measured by laser flash method using an Advance-Riko TC-7000. Triangular prisms were used and minimum angle of deviation method was applied to calculate the refractive index (Möller-Wedel Gmbh, Gonio-Spectrometer Type II). Output power of 50 W laser (1070nm wavelength, cw single mode ytterbium fiber laser manufactured by IPG photonics corp.) was used as a light source to evaluate the thermal lens effect of the materials. Due to thermal lens effect, generally beam shape is slightly deformed after passing through a sample. Change in beam waist of laser beam after passing through each sample was measured as thermal lens effect index by using a beam profiler (Coherent Inc.). In power handling test, pulsed laser (pulse width 50 ps, peak power 0.3 MW, beam spot Φ 0.7 mm, power density 78 MW/cm^2) was irradiated into the optical polished sample at 2 MHz for 7000 h, and inspected the condition of the irradiated sample.

To evaluate Faraday rotation performance, a continuous wave (cw) laser diode (FiberLabs Inc. FPLD-1060-24) was used as an incident laser source (1064nm, max. output 10 mW). Laser was irradiated onto the sample, which is placed between input polarizer and output analyzer made of Glan Thompson prism (GTP). The extinction ratio of the prism was 50 dB. The samples of $(Tb_{0.6}Y_{0.4})_2O_3$ ceramics (5 mm in diameter by 8 mm length) and TGG single crystal (5 mm in diameter by 20mm length, Electro-Optics Technology Inc.) with <111> orientation were used. Each sample was clamped in copper holder and commercial Faraday rotator magnetic housing was used. Nd-B paramagnets (Shin-Etsu Chem. Co.) were used to generate high axial magnetic field. Magnetic field applied to TGG crystal and $(Tb_{0.6}Y_{0.4})_2O_3$ ceramics was 0.98 T and 1.12 T, respectively. A polarization plane of incident laser light was rotated by the Faraday effect because of the magnetic field. The transmitted laser output was measured by using a power meter with respect to each rotation angle of output polarizer ranging from −45 to 135 degree.

Relationship between the Tb Ion Content and Refractive and Thermal Conductivity

Figure S2 shows the relationships between the concentration of Tb ions in TYO ceramics and the refractive index, and the thermal conductivity. The thermal conductivity for Tb = 50~100 % regions is about 3.3–4.6 $Wm^{-1}K^{-1}$, which is comparable to that of the commercial TGG or TAG single crystals.

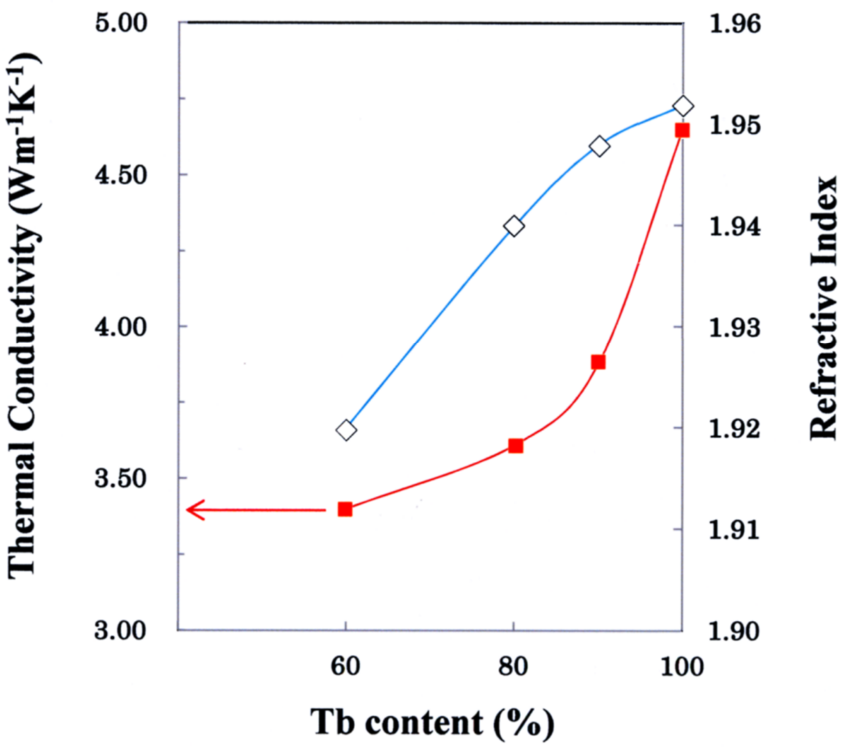

Figure S2. Relationships between the Tb ion concentration and the refractive index, and the thermal conductivity.

Demonstration of Optical Isolator Device Using the TYO Ceramics

Prototype of optical isolator using TYO (Tb-60%) ceramic is shown in Figure S3(a) in comparison with commercial TGG optical isolator. Schematic diagram of general optical isolator is shown in Figure S3(b). It is simply made of an input polarizer (polarized vertically), a Faraday rotator element, and an output polarizer. An AR-coated Faraday rotator element is placed inside an Nd-Fe-B permanent magnet (a hollow cylinder magnet) such that the element is located at the center of light axis. The angle between the input polarizer and the output polarizer is set to 45°. The Faraday rotator is selected to provide a 45° rotation angle with a certain length. As for TGG crystal, it requires 20.0mm length. As for TYO (Tb = 60%) ceramic sample, it requires 8.0mm length in the same magnetic field. As illustrated in Figure S3(c), magnetic flux density decreased with the distance from the center line. Therefore, magnetic field can be more effectively used by placing a shorter element with larger Verdet constant in the case of same magnet house. In other words, as shown in the Figure S3(a), it is possible to produce with smaller magnet house (about half-size by volume) by using the TYO ceramics with larger Verdet constant, leading to miniaturization and low cost at the same time. The features of each Faraday rotator material are summarized in Figure S3(d). If Tb_2O_3 (Tb = 100%) is used, it is certain that the magnet volume can be further reduced as the work is in progress. Principally, when Tb_2O_3 sample with same length as the TGG crystal is used, the required magnet volume can be reduced in accordance with the largeness of the Verdet constant. But for practical use in optical isolator, it cannot be reduced to 1/4 because of the actual distribution of magnetic flux density of magnet housing. For industrial application, issues on downsizing and low cost are very important. By using these ceramic Faraday rotators with highest Verdet constant, it is possible to overcome the weak points of the conventional technology by single crystal materials.

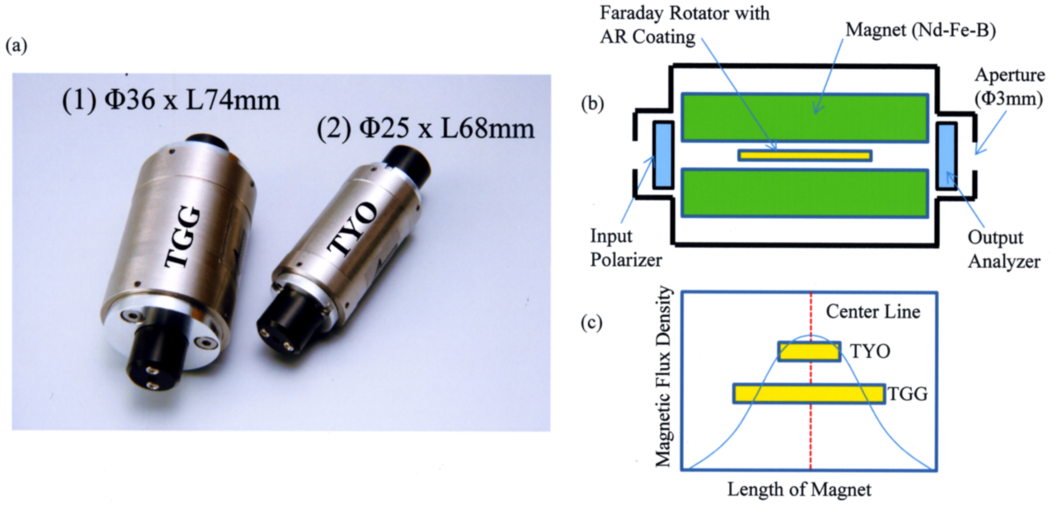

(d)

	(1) Conventional	(2) This Work I	This Work II
Material	TGG Single Crystal	TYO (Tb-60%) Ceramics	Tb_2O_3 Ceramics
Length of Media for 45 deg. Rotation	20.0 mm	8.0 mm	5.1 mm
Normalized Ratio of Magnet Volume	100%	49%	<35%

Figure S3. (**a**) Prototype of optical isolator using TYO (Tb-60%) ceramic in comparison with commercial TGG optical isolator. (**b**) Schematic diagram of optical isolator. (**c**) Magnetic flux distribution inside the magnet house of optical isolator and the position of Faraday rotator sample influenced by the magnetic field. (**d**) Comparison of features of each Faraday rotator material.

Demonstration of Large Aperture Ceramic Isolator for High Power Laser

Good reproducibility and productivity were achieved in this work, which are better than the case of single crystal, with ceramic fabrication technology. For example, in the case of sample with Φ 6 mm × 10 mm dimension, it is possible to produce several thousands to ten thousands of pieces per batch. Samples with 5 mm diameter described above are normally usable for laser power up to 100 W class. For kW class high-power laser operations, Faraday rotator element with large aperture (Φ10~15mm) are required. For application in nuclear fusion and high energy physics in the future, samples with larger aperture (Φ 20–50 mm) will become indispensable. With the invention from this work, it was successful to produce large samples with good transparency (see Figure S4). The work on the development of large scaled samples with improved optical quality is in progress, and it is still necessary to achieve good laser damage performance of large samples higher than the TGG reference. We have confirmed that the laser damage property of the TYO ceramics significantly exceeded the value of TGG but the details of their laser damage properties will be reported in another paper in near future.

Production style of ceramic is different from that of single crystal. In the case of single crystal, a relatively large size crystal is produced and then it is cut and machined to get required smaller size elements. In the case of ceramic, they can be produced in near net shaping to the required size and in large quantity. Therefore, ceramic production style is more favorable than that of single crystal.

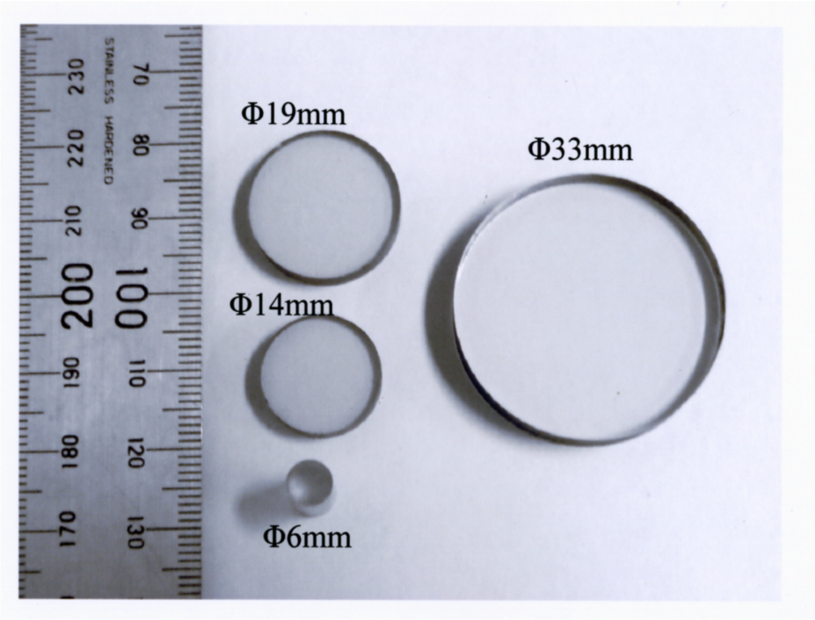

Figure S4. Appearance of large scaled TYO ceramic samples with various aperture sizes.

Figure Captions

Figure S1. (a) External view, and (b) polarized optical microscopic image of $(Tb_{0.5}Y_{0.5})_2O_3$ single crystal.
Figure S2. Relationships between the Tb ion concentration and the refractive index, and the thermal conductivity.
Figure S3. (a) Prototype of optical isolator using TYO (Tb-60%) ceramic in comparison with commercial TGG optical isolator. (b) Schematic diagram of optical isolator. (c) Magnetic flux distribution inside the magnet house of optical isolator and the position of Faraday rotator sample influenced by the magnetic field. (d) Comparison of features of each Faraday rotator material.
Figure S4. Appearance of large scaled TYO ceramic samples with various aperture sizes.

© 2019 by the authors. Submitted for possible open access publication under the terms and conditions of the Creative Commons Attribution (CC BY) license (http://creativecommons.org/licenses/by/4.0/).
